# An At-Risk Population Screening Program for Mucopolysaccharidoses by Measuring Urinary Glycosaminoglycans in Taiwan

**DOI:** 10.3390/diagnostics9040140

**Published:** 2019-10-05

**Authors:** Hsiang-Yu Lin, Chung-Lin Lee, Yun-Ting Lo, Ru-Yi Tu, Ya-Hui Chang, Chia-Ying Chang, Pao Chin Chiu, Tung-Ming Chang, Wen-Hui Tsai, Dau-Ming Niu, Chih-Kuang Chuang, Shuan-Pei Lin

**Affiliations:** 1 Department of Medicine, MacKay Medical College, New Taipei City 252, Taiwan; lxc46199@ms37.hinet.net; 2 Department of Pediatrics, MacKay Memorial Hospital, Taipei 100, Taiwan; wish1001026@gmail.com; 3 Department of Medical Research, MacKay Memorial Hospital, Taipei 100, Taiwan; likemaruko@hotmail.com; 4 MacKay Junior College of Medicine, Nursing and Management, Taipei 100, Taiwan; 5 Department of Medical Research, China Medical University Hospital, China Medical University, Taichung 400, Taiwan; 6 Institute of Biomedical Sciences, MacKay Medical College, New Taipei City 252, Taiwan; 7 Department of Pediatrics, MacKay Memorial Hospital, Hsinchu 300, Taiwan; clampcage@yahoo.com.tw (C.-L.L.); a4964@ms7.mmh.org.tw (C.-Y.C.); 8 Institute of Clinical Medicine, National Yang-Ming University, Taipei 100, Taiwan; 9 Department of Laboratory Medicine, MacKay Memorial Hospital, Taipei 100, Taiwan; andy11tw.e347@mmh.org.tw; 10 Department of Pediatrics, Kaohsiung Veterans General Hospital, Kaohsiung 800, Taiwan; pcchiu@vghks.gov.tw; 11 Department of Pediatric Neurology, Changhua Christian Children’s Hospital, Changhua 500, Taiwan; 128658@cch.org.tw; 12 Department of Biological Science and Technology, College of Biological Science and Technology, National Chiao Tung University, Hsinchu 300, Taiwan; 13 Department of Pediatrics, Chi Mei Medical Center, Tainan 700, Taiwan; whys.tsai@gmail.com; 14 Department of Pediatrics, Taipei Veterans General Hospital, Taipei 100, Taiwan; 15 College of Medicine, Fu-Jen Catholic University, Taipei 100, Taiwan; 16 Department of Infant and Child Care, National Taipei University of Nursing and Health Sciences, Taipei 100, Taiwan

**Keywords:** cross-specialty collaboration, glycosaminoglycans, high-risk screening, liquid chromatography/tandem mass spectrometry, mucopolysaccharidosis

## Abstract

Background: The mucopolysaccharidoses (MPSs) are a group of rare lysosomal storage disorders characterized by the accumulation of glycosaminoglycans (GAGs) and which eventually cause progressive damage to various tissues and organs. We developed a feasible MPS screening algorithm and established a cross-specialty collaboration platform between medical geneticists and other medical specialists based on at-risk criteria to allow for an earlier confirmative diagnosis of MPS. Methods: Children (<19 years of age) with clinical signs and symptoms compatible with MPS were prospectively enrolled from pediatric clinics between July 2013 and June 2018. Urine samples were collected for a non-specific total GAG analysis using the dimethylmethylene blue (DMB) spectrophotometric method, and the quantitation of three urinary GAGs (dermatan sulfate (DS), heparan sulfate (HS), and keratan sulfate (KS)) was performed by liquid chromatography/tandem mass spectrometry (LC-MS/MS). The subjects with elevated urinary GAG levels were recalled for leukocyte enzyme activity assay and genetic testing for confirmation. Results: Among 153 subjects enrolled in this study, 13 had a confirmative diagnosis of MPS (age range, 0.6 to 10.9 years—three with MPS I, four with MPS II, five with MPS IIIB, and one with MPS IVA). The major signs and symptoms with regards to different systems recorded by pediatricians at the time of the decision to test for MPS were the musculoskeletal system (55%), followed by the neurological system (45%) and coarse facial features (39%). For these 13 patients, the median age at the diagnosis of MPS was 2.9 years. The false negative rate of urinary DMB ratio using the dye-based method for these 13 patients was 31%, including one MPS I, two MPS IIIB, and one MPS IVA. However, there were no false negative results with urinary DS, HS and KS using the MS/MS-based method. Conclusions: We established an at-risk population screening program for MPS by measuring urinary GAG fractionation biomarkers using the LC-MS/MS method. The program included medical geneticists and other medical specialists to increase awareness and enable an early diagnosis by detecting MPS at the initial onset of clinical symptoms.

## 1. Introduction

The mucopolysaccharidoses (MPSs) are a group of rare lysosomal storage disorders caused by the deficiency of specific enzymes that catalyze the stepwise degradation of glycosaminoglycans (GAGs). Currently, 11 enzymes are known to be involved in the catabolism of dermatan sulfate (DS), heparan sulfate (HS), keratan sulfate (KS), chondroitin sulfate (CS), and hyaluronic acid. Specific enzyme deficiency leads to the accumulation of GAGs in cells and eventually causes progressive damage to various tissues and organs [1]. The clinical presentations of MPS include coarse facial features, developmental delay, corneal clouding, adenotonsillar hypertrophy, hearing loss, upper airway obstruction, pulmonary function impairment, obstructive sleep apnea, cardiovascular disease, hepatosplenomegaly, short stature, joint stiffness, and skeletal deformities (dysostosis multiplex). The clinical signs and symptoms in these patients are chronic and progressive, and may present from early to late childhood or even in early adulthood, and the severity and prognosis vary among different types with a wide spectrum of clinical severity [2,3,4,5,6,7,8,9,10]. All types of MPS exhibit autosomal recessive inheritance except MPS II (Hunter syndrome), which is transmitted in an X-linked recessive mode and thus predominantly affects males. The incidence of MPS in different populations ranges from 1.9 to 4.5 per 100,000 live births. In Taiwan, this has been estimated to be 2.04 per 100,000 live births [11].

A precise diagnosis of MPS disorders is traditionally made through three consecutive analyses: the quantification of urinary GAGs, two-dimensional electrophoresis (2-D EP) qualitative examination, and leukocyte enzyme activity assay [12,13]. Urinary GAG quantitative tests can be used as a diagnostic screening device for MPS; however, they cannot be used to determine a specific MPS type. The dimethylmethylene blue (DMB) spectrophotometry method is broadly used in most biochemical genetics laboratories; however, it involves a non-specific total GAG assay which can cause both false positive and false negative results, especially in patients with MPS III and IV [13,14]. Two-dimensional EP is the most commonly used method to identify specific types of MPS; however, it is laborious, and its interpretation is subjective and ambiguous, making the diagnosis unreliable. To resolve the limitations of these first-line screening methods for MPS, the liquid chromatography/tandem mass spectrometry (LC-MS/MS) method has been used to identify MPS subgroups, and it has been shown to be an accurate and reliable method [15,16,17,18].

The major therapies for MPS disorders include enzyme replacement therapy (ERT) and hematopoietic stem cell transplantation (HSCT). ERT is now available for MPS I, II, IVA, VI, and VII, and it has been demonstrated to remarkably reduce urinary GAG levels and substantially improve endurance, joint mobility, physiological activities, and quality of life [19,20,21,22,23,24,25,26,27,28]. HSCT is currently the only treatment to prevent progressive neurodegenerative disorders in MPS I, II, VI, and VII [29]. Some developing therapeutics for MPS disorders are currently in clinical trials, including substrate reduction therapy, chaperone therapy, and gene therapy [30,31]. Previous studies have indicated that early treatment may contribute to a better clinical outcome [32,33,34].

Since MPS disorders are rare, multisystemic and progressive diseases with subtle signs and symptoms at the beginning of the natural course, making an early diagnosis can be a challenge for first-line health care professionals. Tracing back the medical history, the patients are usually brought to miscellaneous medical specialists due to diverse manifestations before the confirmative diagnosis of MPS [35,36,37]. In Taiwan, there is insufficient awareness of MPS, which can lead to a delay in the diagnosis or even misdiagnosis with other disorders, and thus these patients often receive inappropriate management. Identifying and understanding the early signs and symptoms of this disease may allow for an early diagnosis and timely appropriate treatment [38,39,40,41]. Therefore, the purpose of this study was to develop a feasible MPS screening algorithm and establish a cross-specialty collaboration platform between medical geneticists and other medical specialists based on at-risk criteria by measuring urinary GAG fractionation biomarkers using the LC-MS/MS method to allow for an earlier confirmative diagnosis of MPS.

## 2. Materials and Methods

### 2.1. Study Population

Children (<19 years of age) with clinical signs and symptoms raising the suspicion of MPS were prospectively enrolled between July 2013 and June 2018 from the pediatric clinics of six medical centers in Taiwan, including Taipei MacKay Memorial Hospital, Hsinchu MacKay Memorial Hospital, Kaohsiung Veterans General Hospital, Taipei Veterans General Hospital, Chi Mei Medical Center, and Changhua Christian Children’s Hospital. The health care professionals were asked to record the clinical signs and symptoms that raised the suspicion of MPS when they sent the urine samples to our laboratory (Table 1). Urine samples were randomly collected in sterile plastic urine containers (polyethylene; Nalge Nunc International, USA) with screw caps and stored in a refrigerator at 4 °C one night before urinary biochemistry analysis or stored in freezer at -20 °C for long-term storage. Urine creatinine was measured using an enzymatic method (Beckman DXC-880i) at the Department of Clinical Laboratory Medicine, MacKay Memorial Hospital.

#### 2.1.1. Ethics Approval and Consent to Participate

All procedures followed were in accordance with the ethical standards of the responsible committee on human experimentation (institutional and national) and with the Declaration of Helsinki of 1975, as revised in 2000. The Institutional Review Board of MacKay Memorial Hospital approved this study, and written informed consent was obtained from all of the patients or their parents who were included in the study.

#### 2.1.2. Consent for Publication

Written informed consent for publication was obtained from all of the patients or their parents who were included in the study.

### 2.2. First-Line Biochemistry Examinations and the Confirmative Methods

#### 2.2.1. DMB Method

In this study, non-specific total GAG analysis was applied using the dye-based spectrophotometry method reported by de Jong J.D. et al. [42,43]. GAGs are determined quantitatively in urine by reaction with the dye dimethylmethylene blue (DMB) in a reaction, and the colour is measured rapidly at a wavelength of 520 nm. The DMB/creatinine ratio, which gives an estimation of the GAG concentration in urine, is age-dependent, and thus the DMB/creatinine ratio can be used as an MPS referential diagnosis but cannot be used for MPS type determination. The higher DMB/creatinine ratio comes mostly from the very young group (infants less than 2 years old); on the contrary, the DMB/creatinine ratio is lower and nearly constant in the adult group (older than 18 years old).

#### 2.2.2. 2-D EP Method

The GAG disaccharide pattern was determined by 2-D EP according to the method proposed by Hopwood et al. [44] and the protocol issued by Willink Biochemical Genetics Unit, Royal Manchester Children Hospital, Pendlebury, Manchester, UK. The 2-D EP method is reliable and specific and can provide a very good separation of GAG components. Two-dimensional EP is the most common and feasible method used for MPS type determination, which is the basis of MPS confirmation.

#### 2.2.3. LC-MS/MS of DS, HS, and KS

The urine quantification of three GAG-derived disaccharides (dermatan sulfate (DS), heparan sulfate (HS), and keratan sulfate (KS)) was performed using the MS/MS-based method. In our study, the principles of methanolysis (chemical hydrolysis) [16,17,45] and keratanase II (specific enzymatic digestion) [46,47,48] were applied separately for the quantification of individual GAG-derived disaccharides including DS and HS, as well as KS by tandem mass spectrometry assay, respectively. The validation accuracy of the MS/MS-based method which we applied was good in that the accuracy (%) of this method was 101.1 (±6.4), 103.3 (±8.1), and 103.9 (±8.3) for DS, HS, and KS, respectively. The intra- and inter-assay precisions (coefficient of variation; CV (%)) were excellent, as the CV (%) values of individual DS, HS, and KS were all less than 10%. The recoveries of this LC-MS/MS assay were 94.3% for DS, 95.1% for HS, and 94.2% for KS. In addition, the linearity of DS (ranging from 12.5 to 200 μg/mL), HS (ranging from 3.125 to 50 μg/mL), and KS (ranging from 31.25 to 1000 μg/mL) was calculated individually, and the correlation coefficients (r) were 0.9972, 0.9969, and 0.9992, respectively. Based on the results detected by the LC-MS/MS method, the quantities of the affected GAGs are distinctive for MPS type determinations. The results fully matched those examined by the 2-D EP method and correspond well with the leukocyte enzyme assay.

As we are aware, KS is either of two glycosaminoglycans (I and II), consisting of repeating disaccharides units of *N*-acetylglucosamine (GlcNAc-6-sulfate) and galactose in the β-1, 4 linkage. KS are present in the cornea, cartilage, and bone. The designations KS I and KS II were originally assigned on the basis of the tissue type from which the keratan sulfates were isolated. KS I was isolated from corneal tissue and KS II from skeletal tissue. According to the conclusion reported by Shimada T. et al., “the level of di-sulfated KS and its ratio to total KS can distinguish control subjects from patients with MPS II, IVA, and IVB, indicating that di-sulfated KS can be a novel biomarker for these disorders.” [49]. Thus, uKS should be measured with both mono- and di-sulfated KS levels in urine; however, we have only shown the quantitative values of mono-sulfated KS in urine in this study.

#### 2.2.4. Enzyme Activity Measurements

MPS enzyme activity measurements include MPS I (β-iduronidase; IDUA) [50,51], II (iduronate-2-sulfate sulfatase; IDS) [51,52], IIIB (α-*N*-acetylglucosaminidase; NAG) [53], IVA (*N*-acetylgalactosamine-6-sulfatase; GALNS) [54], and VI (Arylsulfatase B; ARSB) [55]. Leukocyte isolation from 2–3 mL EDTA blood and protein determination are required prior to performing the enzyme assay. Proteins are determined using Coomassie Plus protein assay reagent (Pierce). The assay for individual enzyme activity is carried out using 4-methylumbelliferyl substrate. The enzyme activity is proportional to the amount of liberated fluorescence detected (excitation, 365 nm; emission, 450 nm) with a Victor Nivo^TM^ Multimode plate reader (Perkin Elmer). The enzyme activity is expressed as μmol enzyme activity/g protein/hour. An individual enzyme’s activity which is 5% lower than that of the normal population is defined as being a marked reduction in terms of the activity of that enzyme.

All urine samples were stored at -20 °C prior to analysis [12,17,18]. The subjects with elevated urinary GAG levels by the DMB spectrophotometry method and abnormal GAG-derived disaccharide patterns by 2-D EP, and elevated urinary DS, HS, or KS levels by the LC-MS/MS method, were recalled for specific enzymatic activity assays in leukocytes by fluorometry for a confirmative diagnosis. Elevated levels of DS and HS indicated MPS I (alpha-iduronidase) and MPS II (iduronate-2-sulfatase), an elevated level of HS indicated MPS III (heparan *N*-sulfatase in type A, alpha-*N*-acetylglucosaminidase in type B, acetyl CoA-alpha-glucosaminide acetyltransferase in type C, and *N*-acetylglucosamine 6-sulfatase in type D), elevated levels of KS and CS indicated MPS IVA (galactose-6-sulfate sulfatase), an elevated level of KS only indicated MPS IVB (beta-galactosidase), an elevated level of DS indicated MPS VI (arylsulfatase B), and elevated levels of DS, HS, and CS indicated MPS VII (beta-glucuronidase). The patients with an enzyme activity <5% of the mean of normal population underwent genetic testing and counseling. The study protocol was approved by the Ethics Committee of MacKay Memorial Hospital, and written informed consent was provided by a parent of the children and from the patients themselves if they were over 18 years of age.

### 2.3. Normal Reference Values of Urinary GAGs

In our previous study, the normal reference values of the three urinary GAGs (DS, HS, and KS) using the LC-MS/MS method, and the non-specific total level of urinary GAGs (DMB/creatinine ratio) using the dye-based method, were determined from 221 urine samples of healthy controls. The reference values were divided into five age groups as follows: 0–1 years, 1–3 years, 4–9 years, 10–17 years, and >18 years of age. The normal reference ranges of DS, HS, and KS were consistent among the five age groups; however, the normal reference ranges of the DMB ratio decreased as the age of the normal controls increased [56].

### 2.4. Clinical Characteristics of Patients with a Confirmative Diagnosis of MPS

For patients with a confirmative diagnosis of MPS, we recorded the clinical characteristics including the MPS type, gender, urinary DS, HS, KS and DMB ratio for which a medical specialist referred the patient for testing, and the existence of clinical manifestations at the time of diagnosis including the involvement of the musculoskeletal system, ophthalmological system, respiratory system, otorhinolaryngological system and neurological system, and cardiac disease, hernia, hepatosplenomegaly, coarse face, hearing impairment, family history of MPS, and whether they had received surgery before the diagnosis.

### 2.5. Statistical Analysis

All results were analyzed using descriptive statistics, including numbers and percentages for categorical variables, and mean, median, and range (minimum and maximum values) for continuous variables. The results were presented as mean ± standard deviation unless otherwise indicated. False negatives were defined as MPS patients with normal results of urinary DMB ratio or urinary DS, HS and KS levels.

## 3. Results

**Figure 1:**
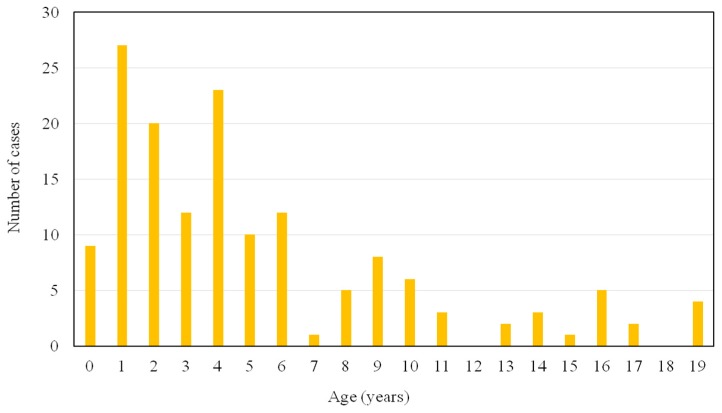
The age distribution of the 153 urine samples collected in this study.

Figure 1 shows the age distribution of the 153 subjects who were enrolled in this study. One hundred and thirteen subjects (74%) were between 0–6 years of age. Forty urine samples sent to our laboratory for at-risk screening for MPS were delivered by pediatric endocrinologists (26%), followed by pediatric neurologists (25%) and general pediatricians (22%) (Table 2). The major signs and symptoms according to the involved system recorded by health care professionals at the time of the decision to test were the musculoskeletal system (55%), followed by the neurological system (45%) and coarse facial features (39%) (Table 3). Twenty-two subjects had elevated urinary GAG levels and abnormal GAG disaccharide pattern by 2-D EP, and elevated urinary DS, HS, or KS levels, and they were recalled for specific enzyme activity assays in leukocytes for a confirmative diagnosis. The patients with an enzyme activity level <5% of the mean of normal population underwent genetic testing. Among them, 13 patients had a confirmative diagnosis of MPS (nine males and four females; age range, 0.6 to 10.9 years; median age, 2.9 years; mean age, 3.4 ± 2.9 years; three with MPS I, four with MPS II, five with MPS IIIB, and one with MPS IVA). Three patients (23%) were identified before 1 year of age, and seven patients (54%) were diagnosed before 3 years of age. The false negative rate of the urinary DMB/creatinine ratio for these 13 patients was 31%, including one MPS I, two MPS IIIB, and one MPS IVA. However, there were no false negative results observed from urinary DS, HS and KS quantifications (Table 4). All 13 patients with MPS had musculoskeletal system involvement, followed by 85% with coarse facial features, 54% with otorhinolaryngological system involvement, 38% with visceromegaly, 38% with hernia, and 38% with neurological system involvement. Two patients diagnosed with MPS II and IVA, respectively, did not have coarse facial features. All five patients with MPS IIIB had speech delay and/or attention deficit. Seven patients (54%) had received surgical procedures before the diagnosis of MPS, including five hernia repairs and three ventilation tube insertions. Six MPS patients were suspected and referred by pediatric neurologists (46%), followed by three patients referred by pediatric orthopedists (23%), and one patient was referred by a pediatric cardiologist, neonatologist, geneticist, and general pediatrician, respectively (Table 5). Intravenous ERT was started in eight patients (Nos. 1–7 and No. 13) following the diagnosis. Patient No. 1 also received HSCT at 1.6 years of age. Patient No. 8 was enrolled in a phase I/II trial of intracerebroventricular enzymatic therapy for MPS IIIB.

## 4. Discussion

To the best of our knowledge, this is the first study to report screening for MPS in an at-risk population in Taiwan by measuring urinary GAG fractionation biomarkers using the LC-MS/MS method, which has been shown to be a powerful and reliable tool for MPS high-risk screening and diagnostic purposes. We established this screening platform with the participation of medical geneticists and other medical specialists to increase awareness and enable an early diagnosis by detecting MPS at the initial onset of clinical symptoms. In this study, three patients (23%) were identified before 1 year of age, and seven patients (54%) were diagnosed before 3 years of age, emphasizing the value of this sign and symptom-based screening program in making an early diagnosis of MPS by raising clinical awareness and educating health care professionals. Colón et al. [57] reported that they diagnosed eight patients with different types of MPS (one with MPS I, one with MPS II, two with MPS IIIA, one with MPS IIIB, two with MPS IVA, and one with MPS VI) by performing a selective screening program for the early detection of MPS from 2014 to 2016. In their cohort, two cases were identified before 1 year of age, and six cases were detected before 3 years of age, which corresponds well with our results. However, compared to their measurement of urinary GAG levels using only the DMB spectrophotometry method and abnormal GAG disaccharide pattern by 2-D EP, we also measured urinary DS, HS, and KS levels using the LC-MS/MS method at the same time. If any of the data were abnormally elevated, the subjects were recalled for specific enzymatic activity assays in leukocytes by fluorometry for a confirmative diagnosis. In this study, the false negative rate of the urinary DMB ratio using the spectrophotometric method for the 13 MPS patients was 31%, including one MPS I, two MPS IIIB, and one MPS IVA. Nevertheless, there were no false negative results of urinary DS, HS and KS using the LC-MS/MS method. Auray-Blais et al. [15] reported that the DMB spectrophotometric method can lead to false negative results because of aggregated formation through electrostatic interactions with albumin, glycoproteins, collagen, and other serum proteins which may modify the physicochemical properties of GAGs, especially in MPS III and IV [14]. In their study, the DMB spectrophotometric method missed the identification of MPS in 30% of their patients (7/23) (one with MPS II, one with MPS III, four with MPS IVA, and one with MPS VI), revealing that this method is relatively unreliable for screening MPS patients, which also corresponds well with our results. Therefore, we recommend that measuring GAG fractionation biomarkers with the LC-MS/MS method is an accurate and reliable method to concurrently quantify urinary levels of DS, HS and KS, and that these biomarkers are more sensitive compared to the traditional DMB ratio using the spectrophotometric method to diagnose MPS, identify subgroups, and screen high-risk populations.

It is well-known that urine GAG is different from blood GAG. The limitation of uKS in its use as a biomarker to prove therapeutic efficacy is distinct. A large amount of literature has revealed that uGAG is not an appropriate biomarker for monitoring therapeutic effects [58]. According to the experimental findings reported by Saville J.T. et al., the concentration of oligosaccharides and the ratio of HS:DS in urine were similar to those observed in the kidney, suggesting that the oligosaccharide storage pattern in urine is a reflection of that in the kidney. Although serum, liver and brain had a similar ratio of HS:DS, which was lower than that seen in the urine and kidney, a distribution of oligosaccharides which ranked from most to least abundant between serum, liver and brain was observed, suggesting that serum more closely reflects the oligosaccharides of the brain and liver and may therefore be a more informative measurement of disease burden than urine [59]. In addition, Kahn et al. [47] and Fujitsuka et al. [60] also stated that blood KS remained high while uKS was reduced during ERT. The fact of the lack of a reduction of blood KS should be notable because this is critical evidence that uKS is ineffective as a biomarker to prove therapeutic efficacy.

Different types of MPS have many similar clinical features and some type-specific manifestations. The severe forms of MPS I and II present with both somatic and cognitive involvement and are characterized by coarse facial features, vision and hearing impairment, recurrent respiratory infections, decreased pulmonary function, obstructive sleep apnea, cardiac disease, inguinal and umbilical hernias, hepatosplenomegaly, spinal cord compression, communicating hydrocephalus, and dysostosis multiplex [56,61]. MPS III manifests as neurological and cognitive impairment and mild somatic involvement [62]. MPS IV presents as short stature, odontoid hypoplasia, ligamentous laxity, joint hypermobility, and skeletal dysplasia [63]. MPS VI is characterized by a purely somatic manifestation similar to MPS I and II without cognitive involvement [1,2,3]. In our study, at the time of diagnosis, all 13 MPS patients had musculoskeletal system involvement, followed by 85% with coarse facial features, 54% with otorhinolaryngological system involvement, 38% with visceromegaly, 38% with hernia, and 38% with neurological system involvement. All five MPS IIIB patients had a speech delay and/or attention deficit. None of the patients had cornea clouding, and one MPS II patient and one MPS IVA patient did not have coarse facial features when they were diagnosed.

Using data from an MPS I registry (*n* = 544), Arn et al. [64] reported that at least one surgery preceded the diagnosis in 36%, 46%, and 63% of the patients with Hurler, Hurler–Scheie, and Scheie syndromes, respectively. In addition, more than one-third (39%) of all patients had hernia repair surgery before they were diagnosed with MPS I. Using data from the Hunter Outcome Survey (*n* = 389), Mendelsohn et al. [65] reported that the majority of patients (57%) underwent at least one surgical intervention before the diagnosis of MPS II, and that 55% and 45% of the patients underwent hernia repair and tympanostomy, respectively, before MPS II was diagnosed. In our cohort, seven patients (54%) received surgical procedures before the diagnosis of MPS, including five with hernia repair and three with ventilation tube insertion, which is consistent with Mendelsohn et al.’s study. These findings indicate that health care professionals need to be aware of the surgical burden of MPS as well as its presenting signs and symptoms and the importance of timely referral for MPS diagnostic testing.

A delayed diagnosis of MPS is often due to referrals from one physician to another. This is mainly because of the rare nature of the disorder, phenotypic heterogeneity, and the broad range of nonspecific early signs and symptoms [35]. As a consequence, in addition to medical geneticists, it is important that general health care professionals are aware of the early signs and symptoms of MPS. In this prospective study, we established an at-risk population screening program for MPS that involved the participation of medical geneticists and other medical specialists, including pediatric endocrinologists, pediatric neurologists, general pediatricians, pediatric rheumatologists, neonatologists, pediatric orthopedists, pediatric cardiologists, and pediatric surgeons. Among the 13 patients diagnosed with MPS, 46% were referred by pediatric neurologists, followed by pediatric orthopedists (23%). Other patients were referred by pediatric cardiologists, neonatologists, geneticists, and a general pediatrician.

Although ERT and HSCT cannot cure MPS disorders, they can improve or lessen the natural progression, and better outcomes may be related to commencing these treatments at a younger age [32,33,34]. The increasing clinical awareness of MPS disease and increased ability to make a confirmative diagnosis has made an earlier diagnosis possible. In this study, eight of the 13 patients diagnosed with MPS started to receive ERT, for which the payments were reimbursed by the Taiwanese National Health Insurance program following international standards. In addition, one MPS I patient also received HSCT, and one MPS IIIB patient enrolled in a phase I/II clinical trial.

## 5. Limitations

Previous studies have reported that elevated GAG levels may be due to conditions unrelated to MPS, including arthritis, diabetes, and mucolipidosis [66,67]. In addition, the small sample size with each type of MPS with a confirmative diagnosis is also a limitation to this study. However, this reflects the rare nature of this genetic disorder. Meanwhile, the degree of disease severity was quite wide, as was the range of age at diagnosis. Thus, further studies with a longer follow-up period and larger cohorts are warranted.

## 6. Conclusions

We established an at-risk population screening program for MPS by measuring urinary GAG fractionation biomarkers using the LC-MS/MS method and included medical geneticists and other medical specialists to increase awareness and enable an early diagnosis by detecting MPS at the initial onset of clinical symptoms. This method appears to be accurate and reliable to simultaneously quantify urinary levels of DS, HS and KS, which have been shown to be more sensitive than the traditional DMB ratio using the spectrophotometric method to diagnose MPS, identify subgroups, and screen high-risk populations. Due to the progressive nature of MPS, initiating ERT or HSCT before the occurrence of irreversible organ damage may contribute to a better clinical outcome. Thus, making an early diagnosis through screening programs for high-risk populations or newborns is very important [57,68,69].

## Figures and Tables

**Table 1:** Signs and symptoms of mucopolysaccharidoses in different organs and systems.

Organs and Systems	Signs and Symptoms
Musculoskeletal system	Skeletal malformations, joint stiffness or hypermobility, short stature, kyphoscoliosis, carpal tunnel syndrome
Neurological system	Psychomotor delay, cognitive delay, behavioral disorders
Face	Coarse facial features
Connective tissue	Hernias
Heart	Valvulopathy, myocardiopathy
Visceromegaly	Hepatomegaly, splenomegaly
Respiratory system	Recurrent respiratory infection, noisy breath sounds
Otorhinolaryngological system	Recurrent otitis media, hearing impairment, recurrent sinusitis, obstructive sleep apnea syndrome
Ophthalmological system	Cornea clouding, retinopathy

**Table 2:** Number and percentage of different specialists who sent the urine sample to our laboratory for at-risk population screening for mucopolysaccharidoses.

Specialist	Number	Percentage
Pediatric endocrinologist	40	26%
Pediatric neurologist	39	25%
General pediatrician	34	22%
Geneticist	17	11%
Pediatric rheumatologist	6	4%
Neonatologist	6	4%
Pediatric orthopedist	5	3%
Pediatric cardiologist	5	3%
Pediatric surgeon	1	1%
Total	153	100%

**Table 3:** Signs and symptoms by different systems recorded by health care professionals at the time of the decision to test the 153 subjects suspected of having mucopolysaccharidosis (MPS).

Signs and Symptoms by Different Systems	Number	Percentage
Musculoskeletal system	84	55%
Neurological system	69	45%
Face	59	39%
Hernias	13	8%
Heart	10	7%
Visceromegalies	9	6%
Respiratory system	7	5%
Hearing system	5	3%
Ophthalmological system	4	3%
Otorhinolaryngological system	4	3%
Family history of MPS	3	2%
Total	267	

**Table 4:** Demographic data, urine glycosaminoglycans (GAG) levels, enzymatic activity, and genotype of the 13 MPS patients at the time of a confirmative diagnosis.

No.	MPS type	Gender	Age at Diagnosis (years)	DS (μg/mL)	HS (μg/mL)	KS (μg/mL)	DMB Ratio (mg/mmol creatinine)	*DMB Reference Range (mg/mmol creatinine)	Enzyme	Enzymatic Activity	Gene	Genotype
1	I	F	0.6	322.1	5.0	3	113.62	<69.15	Alpha-iduronidase	0.3 nmol/mg protein/h	*IDUA*	c.590-7G > A/c.1861C > T
2	I	F	0.7	174	3.9	2.79	159.51	<69.15	Alpha-iduronidase	0.39 nmol/mg protein/h	*IDUA*	c.1192_1194delGAG/c.1634delA, c.1634_1635insGGG
3	I	F	2.9	147.66	21.71	0.7	49.78	<58.82	Alpha-iduronidase	0.87 nmol/mg protein/h	*IDUA*	c.95T > G
4	II	M	0.9	78.69	176.3	0	185.43	<69.15	Iduronate-2-sulfatase	0.1 nmol/mg protein/ 4h	*IDS*	c.137A > C (hemizygous)
5	II	M	3.8	127.69	109.75	6.31	61.29	<58.82	Iduronate-2-sulfatase	0.4 nmol/mg protein/ 4h	*IDS*	c.1122C > T (hemizygous)
6	II	M	6.3	51.8	0	1.51	37.60	<18.20	Iduronate-2-sulfatase	0.2 nmol/mg protein/ 4h	*IDS*	c.1600A > C (hemizygous)
7	II	M	10.9	91.93	384.03	0.22	27.03	<16.81	Iduronate-2-sulfatase	0.86 nmol/mg protein/ 4h	*IDS*	c.1006 + 5G > C (hemizygous)
8	IIIB	M	1.3	0	92.43	3.59	55.35	<58.82	*N*-acetyl-glucosaminidase	0.05 nmol/mg protein/h	*NAGLU*	c.383 + 1G > T/c.1693C > T
9	IIIB	M	1.8	0.3	179.2	0.36	65.23	<58.82	*N*-acetyl-glucosaminidase	0.06 nmol/mg protein/h	*NAGLU*	c.252_253ins19/c.1493T > C
10	IIIB	M	3.4	0	48.59	2.68	47.20	<58.82	*N*-acetyl-glucosaminidase	0.07 nmol/mg protein/h	*NAGLU*	c.383 + 1G > T/c.1693C > T
11	IIIB	M	4.7	0.9	58.1	0.06	22.52	<18.20	*N*-acetyl-glucosaminidase	0.04 nmol/mg protein/h	*NAGLU*	c.926A > G/c.1241A > G
12	IIIB	F	5.0	0.14	11.54	0.01	55.85	<18.20	*N*-acetyl-glucosaminidase	0.2 nmol/mg protein/h	*NAGLU*	c.1693C > A/c.1693C > A (homozygous)
13	IVA	M	1.5	0.04	0.01	90.28	45.27	<58.82	Galactose-6-sulfate sulfatase	0.3 nmol/mg protein/h	*GALNS*	c.953T > G/c.1567T > G

GAG, glycosaminoglycans; MPS, mucopolysaccharidosis; DS, dermatan sulfate; HS, heparan sulfate; KS, keratan sulfate; DMB, dimethylmethylene blue. Reference range: DS < 0.43 μg/mL, HS < 0.46 μg/mL, KS < 7.90 μg/mL. *Reference ranges of DMB are age dependent: 0–1 year, <69.15; 1–3 years, <58.82; 4–9 years, <18.20; 10–17 years, <16.81; >18 years, <12.75 mg/mmol creatinine [42].

**Table 5:** Clinical characteristics of the 13 MPS patients at the time of a confirmative diagnosis.

No.	MPS type	Gender	Age at Diagnosis (years)	Referring Specialist	Musculoskeletal System	Ophthalmological System	Heart	Neurological System	Hernias	Visceromegaly	Face	Respiratory System	Otorhinolaryngological System	Hearing System	Family History of MPS	Surgeries before a Confirmative Diagnosis
1	I	F	0.6	Neonatologist	Y	N	N	N	Y	N	Y	Y	Y	N	M	Herniorrhaphy, supraglottoplasty for laryngomalacia, tracheostomy
2	I	F	0.7	Pediatric orthopedist	Y	N	N	N	N	N	Y	N	N	N	N	None
3	I	F	2.9	Pediatric cardiologist	Y	N	Y	N	N	Y	Y	Y	N	N	N	None
4	II	M	0.9	Geneticist	Y	N	N	N	Y	Y	Y	N	N	N	N	Surgery for spina bifida, herinorrhaphy
5	II	M	3.8	Pediatric orthopedist	Y	N	N	N	Y	N	Y	N	N	N	N	Herinorrhaphy
6	II	M	6.3	Pediatric neurologist	Y	N	N	N	Y	N	N	Y	Y	Y	N	Ventilation tube insertion, tonsillectomy, adenoidectomy, herniorrhaphy
7	II	M	10.9	Pediatric neurologist	Y	N	Y	N	Y	Y	Y	N	Y	N	N	Herinorrhaphy
8	IIIB	M	1.3	Pediatric neurologist	Y	N	N	Y	N	Y	Y	N	Y	Y	Y	None
9	IIIB	M	1.8	General pediatrician	Y	N	N	Y	N	N	Y	N	N	N	N	None
10	IIIB	M	3.4	Pediatric neurologist	Y	N	N	Y	N	Y	Y	N	Y	N	Y	None
11	IIIB	M	4.7	Pediatric neurologist	Y	N	Y	Y	N	N	Y	Y	Y	N	N	Ventilation tube insertion
12	IIIB	F	5.0	Pediatric neurologist	Y	N	N	Y	N	N	Y	N	Y	Y	N	Ventilation tube insertion
13	IVA	M	1.5	Pediatric orthopedist	Y	N	N	N	N	N	N	N	N	N	N	None

MPS, mucopolysaccharidosis; M, male; F, female; Y, yes; N, no.

## Abbreviations

MPS	Mucopolysaccharidosis
GAGs	Glycosaminoglycans
DS	Dermatan sulfate
HS	Heparan sulfate
KS	Keratan sulfate
CS	Chondroitin sulfate
2-D EP	Two-dimensional electrophoresis
DMB	Dimethylmethylene blue
LC-MS/MS	Liquid chromatography/tandem mass spectrometry
ERT	Enzyme replacement therapy
HSCT	Hematopoietic stem cell transplantation
